# Non-surgical Management of Post-traumatic Elbow Stiffness Following a Distal Humerus Fracture: A Case Report

**DOI:** 10.7759/cureus.95069

**Published:** 2025-10-21

**Authors:** Rohit Gulati, Jyotsna Agarwal, Parveen Kaur

**Affiliations:** 1 Pain Management, Nivaan Care, Delhi, IND; 2 Medical Intensive Care Unit, Rajiv Gandhi Cancer Hospital and Research Institute, Delhi, IND

**Keywords:** adhesions, elbow stiffness, fibrosis, hydrodissection, mobilization therapy

## Abstract

This case study examines the non-surgical treatment of post-traumatic elbow stiffness following a distal humerus fracture. A 25-year-old male patient presented with significant pain and a severe restriction in elbow flexion nine months after his injury. The patient had a delayed presentation to the specialist, having previously undergone an unsuccessful closed reduction and manipulation by a local health care provider. The patient received a hydro-dissection procedure using a mixture of corticosteroids, hyaluronidase, and local anesthesia. Within three days of the treatment, he experienced improvement, with his pain-free elbow flexion increasing from 50° to 90°. The patient also reported a better quality of life and improved independence in daily activities. This case highlights hydro-dissection as a potential, minimally invasive, and effective alternative for treating synovial thickening, especially in situations with delays in surgical management or even as a treatment option to avoid surgery.

## Introduction

Synovial thickening and capsular fibrosis are common pathological responses after joint trauma and frequently contribute to elbow stiffness with resultant functional impairment [1,2]. Current clinical management of post-traumatic elbow stiffness is dominated by mechanical or structural approaches such as surgical capsulectomy and arthrolysis (open or arthroscopic), together with intensive physiotherapy and structured rehabilitation programs [1,2]. Several experimental approaches targeting inflammatory or fibrotic pathways have been explored in preclinical models or in inflammatory arthritides. Examples include the use of mast-cell stabilizers such as ketotifen, which has shown reduction in joint capsule fibrosis in rabbit models of post-traumatic contracture [3]; intra-articular relaxin-2, demonstrated to alleviate shoulder arthrofibrosis and reverse established fibrosis when delivered via sustained-release microparticles [4,5]; RNA interference-based strategies aimed at downregulating fibrotic or inflammatory gene expression in arthritic models [6]; and anti-TNF biologics, which have been shown to reduce synovial inflammation and remodeling in spondyloarthropathy [7].

These therapies, while promising in their respective experimental or disease-specific contexts, remain investigational for post-traumatic synovial fibrosis and are not established, routine treatments for elbow arthrofibrosis [1,2].

We describe a case where the patient with post-traumatic elbow stiffness having synovial thickening and joint effusion was managed with hydro-dissection of the joint nine months after the injury. The patient showed marked improvement in pain-free range of motion and resting pain within three days. To our knowledge, limited literature supports such delayed non-surgical management of post-traumatic elbow stiffness in humans.

The etiology of post-traumatic elbow stiffness is multifactorial, involving both soft tissue contractures and bony abnormalities. Capsular thickening, disorganized collagen, cytokine and enzyme changes, and early myofibroblast proliferation driven by the transforming growth factor-beta pathway are central to soft tissue contracture, particularly in the early post-injury phase [8]. Heterotopic ossification, characterized by the formation of mature lamellar bone in non-osseous tissue, creates a mechanical block to motion and is commonly triggered by trauma, burns, or surgery [8]. Extra-articular malunions, especially of the distal humerus, alter the anterior translation necessary for elbow flexion, while intra-articular malunions distort joint congruity and can cause periarticular fibrosis and ulnar nerve dysfunction [8]. Non-unions, whether intra- or extra-articular, contribute to stiffness through articular distortion, adhesions, and instability. Lastly, posttraumatic loss of articular cartilage may lead to arthrosis, with distal humerus fractures being particularly associated with long-term degenerative changes [8]

In this context, our successful use of hydro-dissection with corticosteroid, hyaluronidase, and local anesthetic suggests that such an approach may serve as a viable, less invasive, non-surgical alternative for patients with post-traumatic elbow stiffness.

## Case presentation

**Figure 1:**
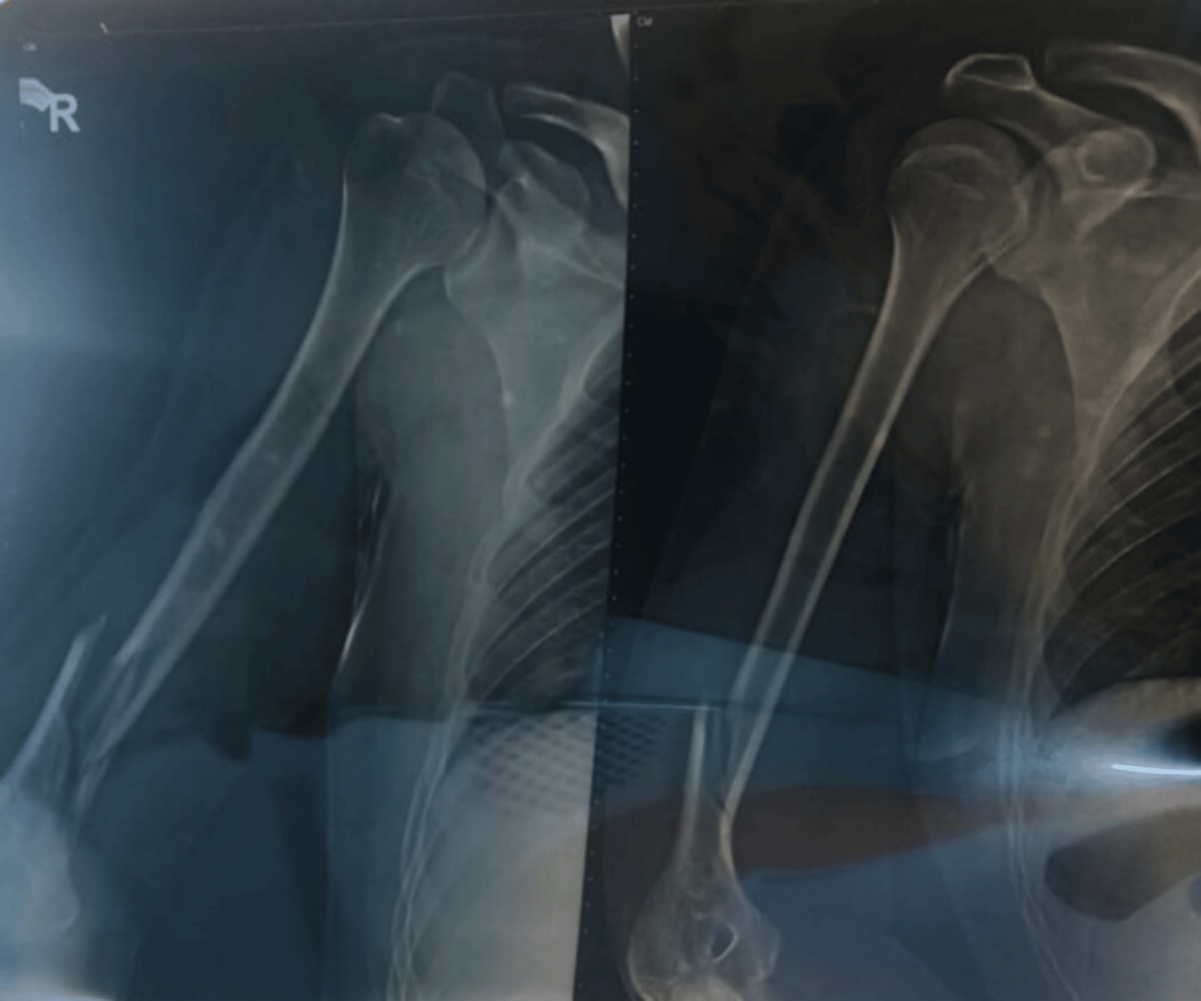
Radiographic image depicting a fracture of the right humerus.

A 25-year-old male presented with post-traumatic restriction of right elbow flexion. The patient had a road traffic accident nine months back, leading to a fracture of the lower one-third of the right humerus (Figure 1).

**Figure 2:**
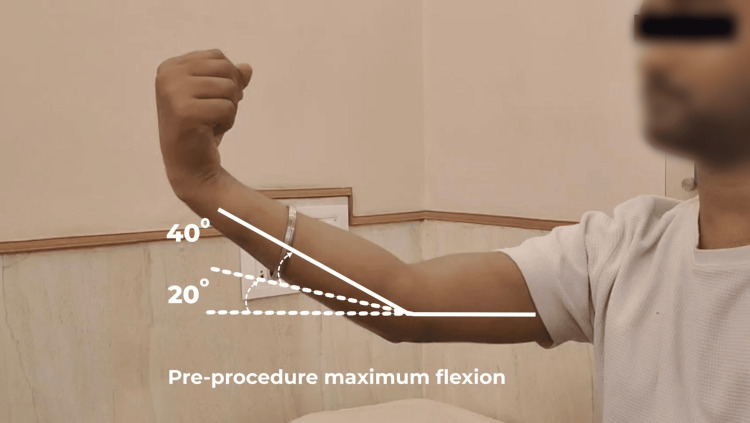
Pre-procedure maximum flexion

The patient, being in a remote location, could not undergo any surgical intervention. He received closed reduction and elbow immobilization for up to 12 weeks. Following this, he started physiotherapy for the right upper limb, and the patient also used to do self-massage of the right elbow region. On examination, the right elbow showed an active range of motion from 20° to 40° and a passive range of motion from 20° to 50°. The patient was unable to perform routine activities requiring hand-to-face movement, such as brushing teeth, eating, or combing hair, and due to pain on elbow flexion, he was also unable to lift his baby in his lap (Figure 2).

Any attempt to flex beyond 40° was accompanied by severe pain, Visual Analogue Scale (VAS) 8-9 out of 10. The patient also had resting pain of VAS 3 out of 10. Muscular atrophy of the right biceps and triceps was noticed, with the right arm showing 4 cm less mid-arm circumference as compared to the left. The patient was previously a regular gym-goer and had no comorbidities or any relevant past history.

**Figure 3:**
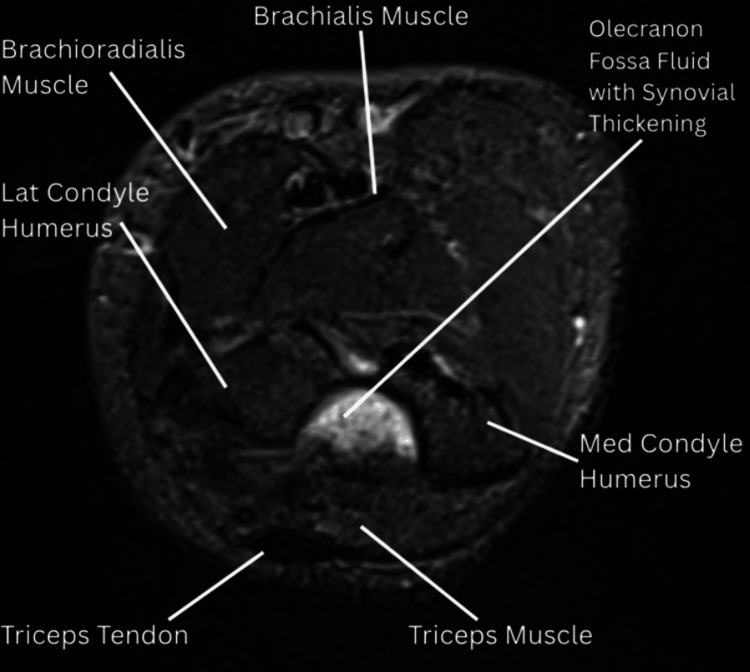
MRI (pre-treatment): axial proton density fat-suppressed (PDFS) image demonstrating mild joint effusion in the olecranon fossa and mild synovial thickening.

MRI imaging of the right elbow, done on the current OPD visit, demonstrated moderate intra- and periarticular fluid accumulation across all joint compartments, with associated synovial thickening and patchy marrow edema, suggestive of moderate synovitis (Figure 3). The injection was administered using the blind technique without USG/CT/MRI guidance.

The patient denied surgical intervention but agreed to a trial of hydro-dissection of the joint. Pre-procedure written informed consent was taken, and lignocaine sensitivity testing was done. Vitals monitoring and aseptic precautions were observed during the procedure. Under local anesthesia, hydro-dissection of the elbow joint and intra-articular and peri-articular soft tissues was done, using 30 ml of drug solution. Hyaluronidase was used as an adjuvant in the hydro-dissection mixture to enzymatically reduce hyaluronic-acid-mediated viscosity and to improve dispersion of the injectate, thereby facilitating mechanical separation of adhesions and enhanced delivery of corticosteroid and local anaesthetic. While this mechanism is biologically plausible and supported by studies of peri-articular hydro-dissection and adjunctive injections, high-quality evidence specifically demonstrating reversal of post-traumatic synovial thickening in the elbow is currently lacking; therefore, we present this as a less-invasive, investigational alternative to surgery in selected patients.

**Figure 4:**
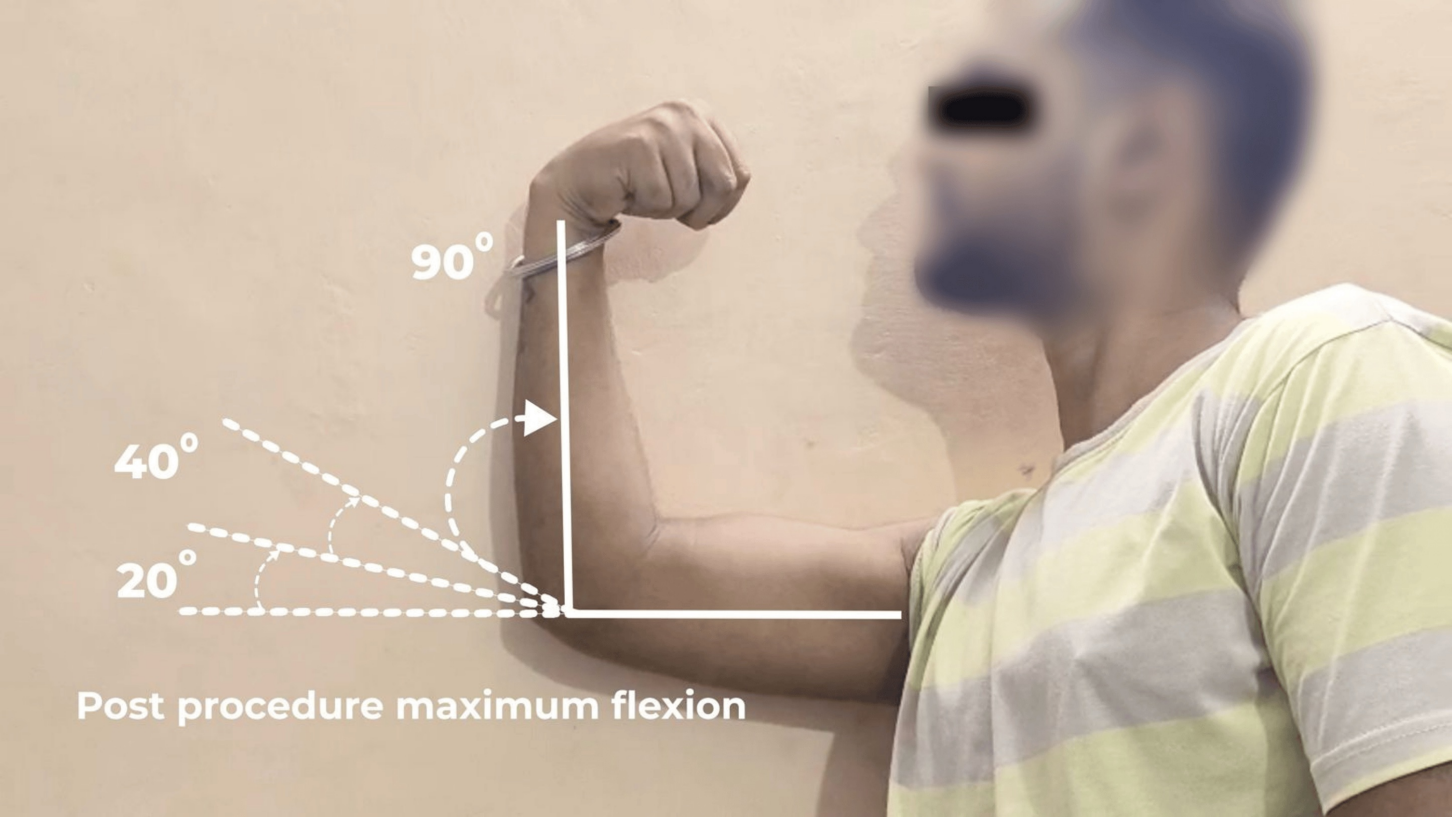
Post-procedure maximum flexion

The drug solution consisted of injection methylprednisolone acetate 80 mg, 1% lignocaine, and 3000 IU hyaluronidase, with normal saline. Within 15 minutes post-procedure, the patient experienced a reduction in elbow stiffness and a reduction in pain on movement. Intermittent cold fomentation was done on the site for two days. Functional improvement was assessed on day three. Significant improvement in range of motion was noted, with pain-free passive flexion-extension from 20° to 90°. Pronation and supination were full and painless (Figure 4).

There was no resting pain at this time. Fifteen days later, a repeat hydro-dissection procedure was done, similar to the first procedure. The dosing was adjusted between the two sittings. In the first procedure, the patient received 80 mg of Depo-Medrol, and in the second sitting (after three weeks) 40 mg of Depo-Medrol was administered. The total dose administered (120 mg) was well within the recommended annual safety limit of 3 mg/kg body weight (≈225 mg for this 75-kg patient). A three-week interval was maintained between the procedures to allow for excretion of the first dose (reported elimination time 12-16 days), thereby ensuring safety.

Minimal improvement was observed after the second procedure. The patient was advised to do gentle physiotherapy exercises. Following the intervention, a comprehensive physiotherapy regimen was initiated under supervision. The detailed physiotherapy protocol is attached in Appendix 1 for reference. At eight weeks post-procedure, the pain-free flexion up to 90° was maintained, and a 20% increase in muscle bulk in the right arm was noted. Restoration of function for daily activities was achieved, as reflected by the patient’s regained ability to perform routine tasks such as brushing, eating, and combing hair. The improvement in elbow flexion also enabled him to comfortably lift and hold his baby, resulting in significant functional and emotional satisfaction.

**Figure 5:**
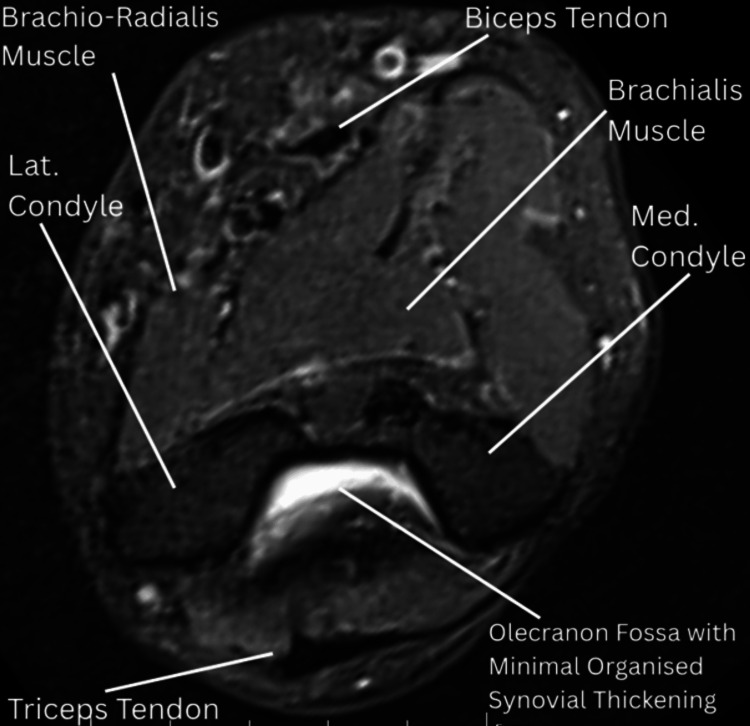
MRI (post-treatment): axial proton density fat-suppressed (PDFS) image demonstrating a marked reduction in diffuse synovial thickening, with only minimal organized residual synovial tissue remaining.

A follow-up (post-treatment) MRI done at eight weeks after the first procedure revealed a reduction in synovial thickening (Figure 5).

## Discussion

This case highlights the potential value of a minimally invasive, non-surgical approach for managing post-traumatic synovial thickening. Ring et al. in 2006 evaluated 46 adults undergoing open elbow capsulectomy and demonstrated a mean improvement of 53° in ulna-humeral motion, with outcomes closely tied to pain and ulnar neuropathy [9]. Their study reinforces surgical release as the standard for refractory elbow stiffness. Similarly, Adithyaa et al. in 2025 reported successful open arthrolysis in a patient with severe post-traumatic stiffness and a flexion arc limited to 30°, secondary to malunion and heterotopic ossification [10].

Hydrodissection (or hydrodilatation) has been reported in other joints and entrapment syndromes with encouraging outcomes. In adhesive capsulitis (frozen shoulder), ultrasound-guided hydrodistention has produced sustained improvements in pain and shoulder range of motion [11-14]. In peripheral nerve entrapment syndromes such as carpal tunnel syndrome, hydrodissection with or without steroid or hyaluronidase adjuncts under ultrasound guidance has been used to release perineural adhesions; randomized controlled trials and systematic reviews suggest it is safe and can improve symptom scores and function over several months [15-17]. These examples support the potential generalizability of hydrodissection approaches to other anatomical sites, while emphasizing that application in post-traumatic elbow stiffness remains exploratory.

The authors emphasize that intra-articular deformities and bony blocks often necessitate open surgical access to restore function. In our case, despite similar mechanical restriction, the absence of major bony obstruction and patient preference enabled a non-surgical approach that offers a promising alternative, potentially disrupting adhesions, reducing inflammation, and enhancing joint mobility. Our patient received two sessions of hydrodissection. Repetitions of the procedure can be individualized as per the patient's presentation and improvement per session. While most clinical literature focuses on surgical management, preclinical models have also explored non-surgical mechanisms of contracture.

Adhesiolysis has been well-documented in the management of post-traumatic stiff elbow, predominantly through surgical or arthroscopic techniques. Open arthrolysis involving adhesiolysis of the capsule and periarticular soft tissues remains an established treatment modality for post-traumatic elbow contracture, enabling substantial restoration of joint mobility [18]. Arthroscopic arthrolysis has also demonstrated favorable outcomes, providing a minimally invasive approach for releasing intra-articular and capsular adhesions while minimizing soft tissue trauma [19]. Additionally, adjunctive strategies such as the intra-articular application of hyaluronan-based anti-adhesion gels during arthroscopic arthrolysis have been explored to mitigate postoperative adhesion recurrence and enhance long-term functional recovery [20]. Complementing these clinical insights, Dunham et al. developed a non-surgical rat model of post-traumatic elbow stiffness, wherein soft tissue injury followed by 42 days of immobilization without pharmacologic intervention led to persistent contracture characterized by capsular fibrosis and myofibroblast proliferation, underscoring the biological basis of post-injury joint stiffness. Our case employed targeted hydro-dissection using corticosteroids, hyaluronidase, and local anesthetic, resulting in rapid and sustained improvement in range of motion within three days. The hydrodissection mixture was intentionally formulated for complementary pharmacologic effects. The corticosteroid (Depo-Medrol) provides potent anti-inflammatory activity, attenuating synovial inflammation and capsular fibrosis; however, repeated use has been associated with potential adverse effects such as local tissue atrophy and systemic absorption-related complications [21]. Hyaluronidase enzymatically reduces hyaluronic acid-mediated viscosity, enhances tissue permeability and drug dispersion, and facilitates adhesiolysis-although its role in post-traumatic elbow stiffness remains investigational, and rare hypersensitivity reactions have been reported [22,23]. The local anesthetic component ensures immediate analgesia, enables comfortable hydrodissection, and supports early mobilization, though its duration of action is short and systemic toxicity can occur if inadvertently administered intravascularly or in excessive doses. Collectively, these agents act synergistically to release adhesions, reduce pain, and promote functional recovery, while emphasizing the need for further studies to validate their combined use in this context.

An increase in flexion from 50° to 90° might appear to be a moderate improvement of 40° flexion; however, it translates into meaningful enhancement in daily activities. An increase in flexion from 50° to 90° might appear to be a moderate improvement of 40° in range of motion; however, it translated into meaningful enhancement in daily activities. The ability to use his right hand, particularly to hold his baby in his lap, gave the patient great satisfaction and joy. This moderate improvement in elbow flexion has led to a remarkable improvement in the patient’s quality of life. There has been no improvement in the minimal restriction in elbow extension (fixed flexion deformity at 20°), and it also appears that further improvement in extension and flexion beyond 90° is not possible non-surgically in this patient. Strengths of this case report include the rapid clinical response, procedural safety, and technical simplicity. 

## Conclusions

This case demonstrates that in carefully selected patients of post-traumatic elbow stiffness, with synovial thickening and no bony obstruction, hydro-dissection of the elbow joint may offer an effective, minimally invasive management technique. In this case, hydro-dissection was performed using a combination of steroid, hyaluronidase, and local anesthetic. Further research in larger cohorts is needed to establish its long-term efficacy, and studies should also explore the feasibility of hydro-dissection as a first-line intervention in such cases, with surgical management reserved for non-responders.

## Appendices

Post-Traumatic Elbow Stiffness - Home Exercise Program

Patient Profile:

- Post distal humerus fracture, treated conservatively

- Post-injection (corticosteroids + hyaluronidase)

- Goal: Increase ROM, reduce stiffness, regain functional mobility

Precautions:

1.Avoid sudden forceful movements

2.Stop if severe pain occurs (>7/10 VAS)

3.Follow pain-free progression

4.Use a towel or strap for assistance if needed
 

1. Phase I - Immediate Post-Injection (Day 1-3)

Goals: Reduce pain, begin gentle motion, prevent adhesions.

**Table 1:** Phase I – Immediate Post-Injection (Day 1–3): Exercise protocol focusing on gentle, pain-free passive and assisted range-of-motion activities initiated to maintain joint mobility and prevent periarticular stiffness.

Exercise	Technique	Reps	Frequency	Description
Gentle Flexion-Extension	Sit with arm supported. Bend and straighten elbow slowly within pain-free range.	10–15	3–5x/day	Picture: Arm on table, moving like “folding a book”
Forearm Supination-Pronation	Elbow at 90°, rotate palm up and down slowly	10	3–5x/day	Picture: Forearm supported on table, palm rotating up/down
Shoulder Scapular Retraction	Pull shoulder blades back, hold 5s	10	3–5x/day	Picture: Seated, hands on lap, squeeze shoulder blades

2. Phase II - Early Mobilization (Day 3-7)

Goals: Gradually increase ROM and reduce stiffness.

**Table 2:** Phase II – Early Mobilization (Day 3–7): Exercise protocol emphasizing gradual transition to active-assisted and active range-of-motion exercises within the pain-free range, aiming to enhance flexibility, reduce periarticular adhesions, and promote functional recovery.

Exercise	Technique	Reps	Frequency	Description
Self-Assisted Flexion	Use opposite hand to lift forearm towards shoulder	10–15	3–4x/day	Picture: Hand of unaffected arm lifting forearm
Self-Assisted Extension	Place hand under forearm, gently push to straighten	10–15	3–4x/day	Picture: Forearm supported, gently pushing to straight
Towel Stretch	Hold towel with both hands, one hand behind back, other over shoulder, slide up/down	10	2–3x/day	Picture: Elbow behind back, other hand holding towel over shoulder

3. Phase III - Active Functional Mobility (Week 2-3)

Goals: Strengthen surrounding muscles, improve functional range.

**Table 3:** Phase III – Active Functional Mobility (Week 2–3): Exercise protocol focusing on progressive strengthening, active range-of-motion, and functional task-oriented movements to restore coordination, endurance, and optimal use of the affected limb in daily activities.

Exercise	Technique	Reps	Frequency	Description
Wall Walk (Flexion)	Place fingers on wall, walk hand up as high as possible	10	2–3x/day	Picture: Hand “walking” up wall
Table Slide (Extension)	Place palm on table, slide forward to straighten elbow	10	2–3x/day	Picture: Arm sliding forward on table
Forearm Rotation with Object	Hold small object (weight 0.5–1kg), rotate palm up/down	10	2–3x/day	Picture: Forearm resting on table, rotating with small weight

4. Phase IV - Strengthening & Endurance (Week 3-6)

Goals: Improve muscle control, endurance, and functional use.

**Table 4:** Phase IV – Strengthening & Endurance (Week 3–6): Exercise protocol emphasizing progressive resistance training, sustained stretching, and endurance-building activities to enhance muscular strength, joint stability, and long-term functional performance of the elbow.

Exercise	Technique	Reps	Frequency	Description
Elastic Band Flexion/Extension	Attach band, bend and straighten elbow against resistance	10–15	2–3x/day	Picture: Band anchored, pulling for flexion/extension
Pronation/Supination with Band	Band in hand, rotate forearm against light resistance	10	2–3x/day	Picture: Elbow at 90°, rotating band slowly
Grip Strength	Squeeze soft ball or putty	10–15	3x/day	Picture: Squeezing ball with hand

5. Self-Mobilization Techniques

a. Tabletop Elbow Flexion Stretch

- Lean forward, forearm on table, let gravity assist flexion.

- Hold 20-30s, repeat 3-5x.

- Picture: Forearm resting on table, elbow bending naturally.

b. Prone Hanger Stretch (Extension)

- Lie prone, elbow off table edge, allow gravity to extend.

- Hold 20-30s, repeat 3-5x.

- Picture: Patient lying on stomach, arm hanging over edge.

c. Self-Assisted Flexion with Towel

- Use towel to gently pull forearm toward shoulder, hold 10-15s.

- Repeat 10x.

- Picture: Towel looped around forearm, pulling with opposite hand.

6. Progression Guidelines

- Move pain-free range first, then gently increase ROM

- Increase repetitions gradually

-Add light resistance (bands/weights) only after 3-4 weeks

-Functional tasks: feeding, grooming, typing can be incorporated as tolerated

7. Outcome Measures

-ROM: Goniometer flexion/extension and pronation/supination

-Pain: VAS

-Function: QuickDASH or Patient-Rated Elbow Evaluation (PREE)

-Strength: manual testing
